# Analysis of morphological parameters of vertebrae in domestic geese and ducks

**DOI:** 10.3389/fvets.2025.1531363

**Published:** 2025-05-09

**Authors:** Jiajia Wang, Zheng Zhang, Kailong Zhou, Xinming Jiang, Dongyan Huang, Zhihui Qian, Lei Ren, Luquan Ren

**Affiliations:** ^1^College of Engineering and Technology, Jilin Agricultural University, Changchun, China; ^2^Key Laboratory of Bionic Engineering, Ministry of Education, Jilin University, Changchun, China

**Keywords:** bird neck, goose neck, duck neck, bone structure parameters, bone surface area

## Abstract

**Background:**

Birds have evolved morphologically diverse neck structures in order to adapt to different foraging styles. Studying avian neck structure and function can not only help us understand their evolutionary processes and ecological roles, but also use them as a research model for modular structure, providing a valuable reference for exploring the morphological and kinematic properties of other organisms.

**Objective:**

By analyzing the neck bone structure of Chinese geese and domestic ducks, referring to the characteristic parameters of the neck bone structure, it provides reference for the structural design of bionic bird neck machinery.

**Methods:**

This study focuses on two representative avian species of the order Anseriformes—geese and ducks—as research subjects to analyze their cervical vertebral structural morphology and investigate characteristic parameters of their cervical skeletal system. This manuscript mainly includes the following contents: structural morphological dimensions of goose and duck vertebrae were manually measured, and characteristic curves were plotted, analysis of their skeletal structural features. Images of the surface structural morphology of goose and duck vertebrae were taken, processed and measured with Image J software. Through comparative analysis of the morphological characteristics of vertebral surface structures in geese and ducks, their patterns were summarized.

**Conclusion:**

The vertebral morphological characteristics of domestic geese and domestic ducks are very similar. With the increase of vertebral length (CL), the fluctuation range of vertebral width (ZW) gradually increases, and the fluctuation trend of vertebral width (ZW) of goose and duck neck is basically the same. The overall changes of the goose cervical CL values were smaller as compared to the duck cervical spine. Furthermore, although geese and ducks differ in cervical spine numbers, both showed functional zoning similar to other birds.

## 1 Introduction

Birds have evolved morphologically diverse neck structures in order to adapt to different foraging styles. Studying avian neck structure and function can not only help us understand their evolutionary processes and ecological roles, but also use them as a research model for modular structure, providing a valuable reference for exploring the morphological and kinematic properties of other organisms (1–5) example, Kambic et al. (6) summarized the range of movement of the bird neck into a three-region model, and found that the joint activity is closely related to the morphological characteristics of the cervical spine, predicting the functional performance through these morphological features. Boas (7) divided the avian cervical spine into three major regions based on the degree of dorsal curvature, and the boundaries between these regions are given by transitional vertebrae with a specific morphology. Knowing the vertebral morphology in each region inferred the movements that might occur.

The avian cervical spine consists of three primary components: muscles, the skeletal system, and nerves. Muscles are the source of locomotor capability; nerves act as intermediaries for signal transmission; and skeletal structural characteristics form the foundation of movement. Among these, the skeletal system includes bones (vertebrae) and joints (saddle-shaped joints between vertebral bodies, as well as articulations between the cranial and caudal articular processes). Birds possess only a single occipital condyle, in contrast to the double occipital condyles of mammals. This single condyle reduces the contact area between the skull and cervical vertebrae, granting the head greater freedom of movement (8–11). Research indicates that birds achieve a wide range of neck motion by combining coordinated intervertebral joints and highly flexible zygapophyseal joints (12–15). Panyutina and Kuznetsov (16) utilized CT scanning to analyze the cervical structure of owls, investigating the relationship between skull flexibility and cervical morphology. They found that the owl’s ability to rotate its head extensively is primarily determined by its skeletal structure. Wang et al. (17) employed dynamic *in vivo* experiments with biplanar X-ray motion capture, revealing that the C4/C5 joint in geese exhibits a larger range of motion compared to other joints, which gradually decrease in mobility. Additionally, these joints can simultaneously perform flexion/extension, lateral bending, and axial rotation. Buchmann and Rodrigues (18) conducted anatomical studies on the cervical spine of aquatic birds, correlating vertebral morphology with foraging strategies through kinematic analysis of cervical joints. Their findings revealed that the avian cervical structure imposes distinct biomechanical constraints: the mid-cervical region primarily facilitates dorsoventral motion, the cervicothoracic junction (base of the neck) permits lateral bending and axial rotation, while the cranial-most segments exhibit polyaxial mobility, enabling near-omnidirectional movement at the head–neck interface.

During daily activities, ducks and geese exhibit remarkable motor agility and fluency, and these motor properties are tightly associated with their unique neck architecture. Anatomical studies show that ducks have 14–15 cervical joints (19, 20), while geese have 17–18 cervical segments (21). Although some studies have explored the neck bone structure of domestic geese and domestic ducks (17, 22, 23), However, the study of the structural characteristic parameters and morphological rules of the neck is still not deep enough. This study conducted anatomical preparations on the cervical vertebrae of four geese and four ducks, employing traditional vernier caliper measurements combined with image processing techniques to quantify the surface morphological parameters of their cervical bones. Subsequent analysis of these parameters elucidated the structural characteristics and articular parameter features of the cervical vertebrae in geese and ducks. By systematically analyzing the morphological characteristic parameters of cervical skeletal structures in domesticated Chinese geese (*Anser cygnoides* domesticus) and ducks (*Anas platyrhynchos* domesticus), this research provides valuable reference data for the structural design of avian-inspired robotic manipulators. The findings offer biomechanical insights applicable to developing articulated robotic arms that mimic avian neck mobility.

## 2 Materials and methods

### 2.1 Test objects

Four specimens of geese (*Anser cygnoides* domesticus) and four ducks (*Anas platyrhynchos* domesticus) with similar age, body weight, and size were selected as test subjects for morphometric analysis of avian vertebral surface structures. Specimen preparation (24): the neck of goose and duck was manually dissected with anatomical tools, with skin, muscle and viscera removed. The wire was inserted into the pillow bone orifice to stabilize the vertebrae (25). The bones were boiled in 5% potassium hydroxide for 2 h and left in the open air for 5 days to enhance maceration. After washing, the bones were soaked in 5% hydrogen peroxide for 3 days to bleach (26).

The cervical spine of geese typically comprises 17–18 vertebrae. Due to their specialized anatomical configuration, the C1 and C2 vertebrae articulating with the cranium primarily facilitate rotational movement rather than sagittal or coronal plane flexion. Their distinct morphological characteristics and wide range of activities render them unsuitable for the quantitative analytical methodology employed in this study. Similarly, the C16 and C17 vertebrae demonstrate significantly reduced mobility compared to C3-C15 segments. These terminal vertebrae function primarily as protective structures at the cervicothoracic junction, exhibiting marked morphological divergence from the C3-C15 vertebrae. Consequently, the C3-C15 segments in geese (corresponding to C3-C12 in ducks) were selected for systematic evaluation of distinctive vertebral structural characteristics.

### 2.2 Parameter definition

**Figure 1:**
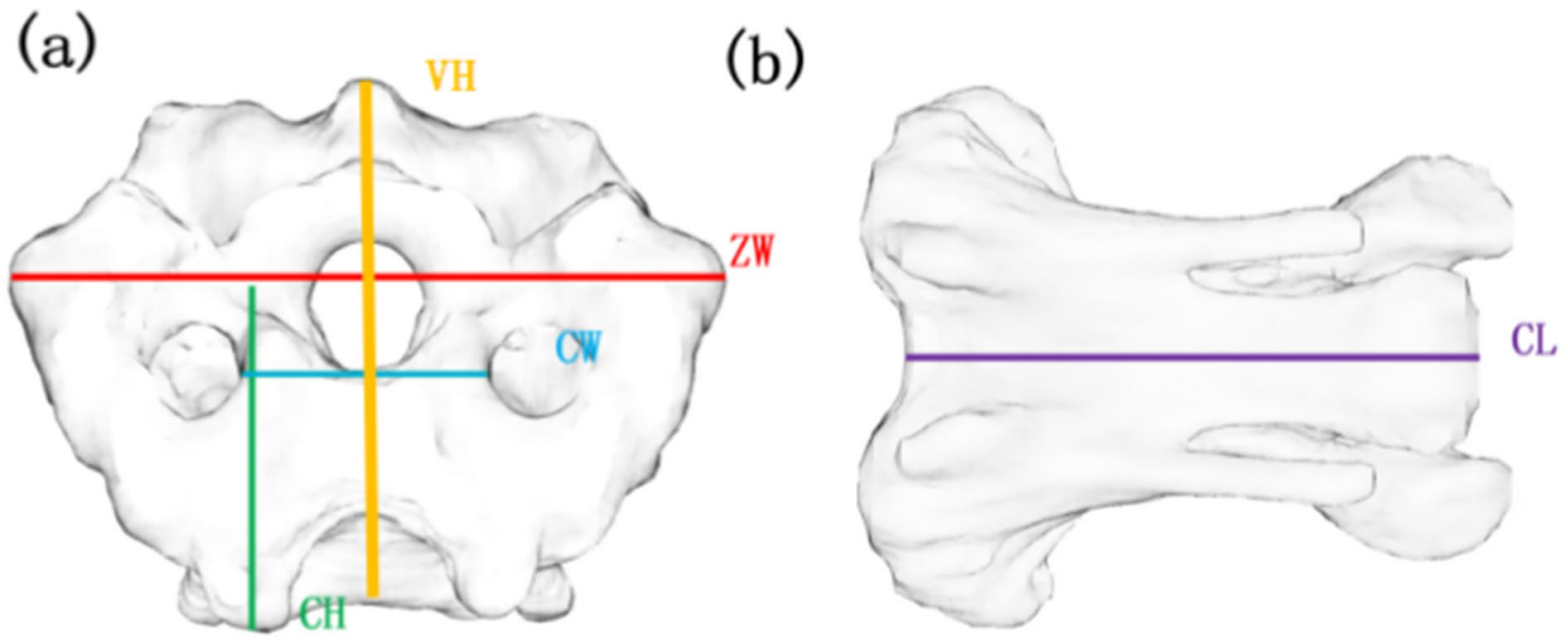
Parameters for measuring cervical bones in birds. **(a)** Front view of C13, **(b)** Side view of C13 VH, vertebral height; ZW, zygapophyseal width; CH, centrum height; CW, centrum width; CL, centrum length.

This study examined the structural properties of avian cervical vertebrae by measuring vertebral height (VH), zygapophyseal width (ZW), center height (CH), center width (CW), and center length (CL), as illustrated in Figure 1. VH refers to the distance between the inferior and superior aspects of the bird’s cervical vertebrae. ZW denotes the distance between the two synchondroses at the caudal terminus of the bird’s cervical vertebrae. CH indicates the height at the caudal end of the bird’s cervical vertebrae. CW represents the width of the skeleton at the caudal end of the bird’s cervical vertebrae, while CL signifies the overall length of the bird’s cervical vertebrae.

**Figure 2:**
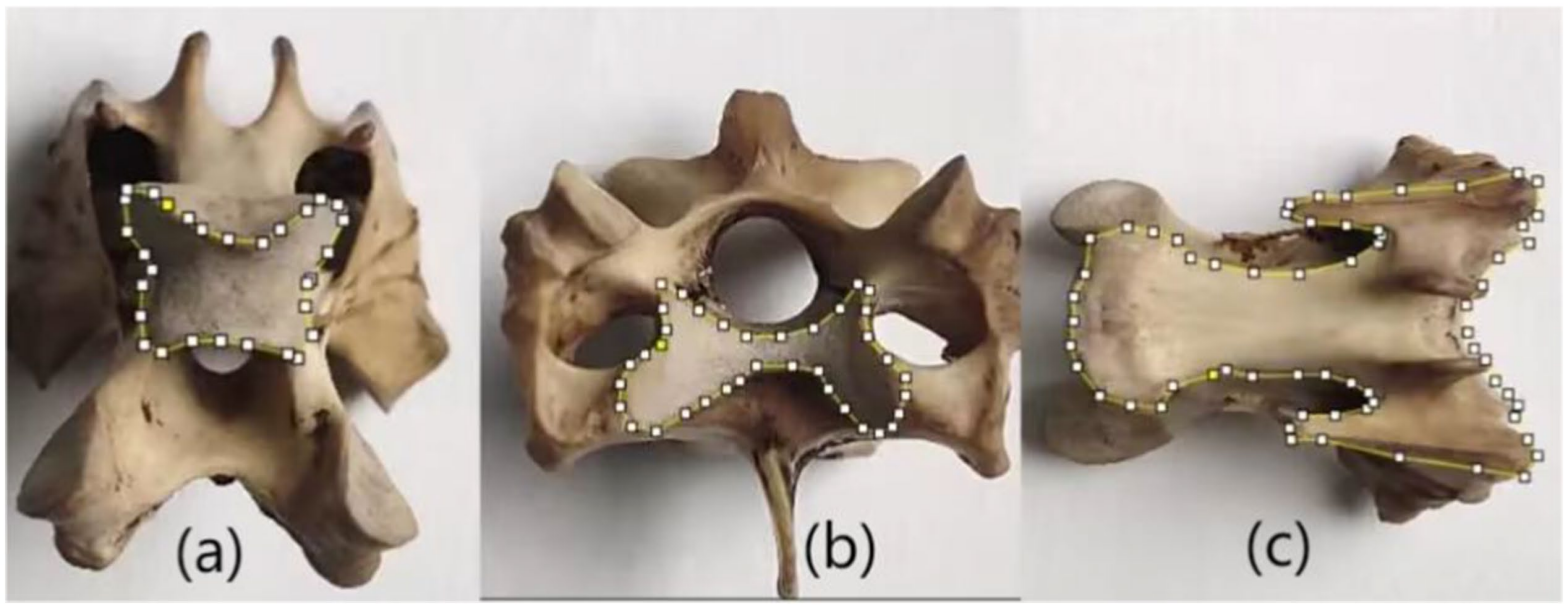
Three typical vertebral surfaces. **(a)** Saddle-shaped joint surface **(b)** M-shaped joint surface **(c)** fan-shaped surface.

Furthermore, the avian cervical spine primarily consists of heterocoelous vertebrae connected by saddle-shaped articulations. This unique joint morphology enables large-angle flexion while maintaining articular stability, thereby facilitating both terrestrial locomotion and aerial maneuvers. Quantification of the saddle joint contact area provides critical insights into understanding and evaluating the specialized bending mechanics and stabilized motion of the cervical vertebrae. Consequently, this study focuses on characterizing the dimensional parameters of these saddle joint surfaces. The ventral osseous surface serves as a primary morphological representation of cervical vertebrae and constitutes a major component of vertebral size. However, no existing literature has investigated the biomechanical influence of ventral surfaces on motion dynamics. In this study, the ventral surface area is primarily employed for bone surface area normalization. The main measuring methods are as follows: The edges of the three typical surfaces described above are manually drawn using Image J software, as shown in Figure 2. In the three typical surfaces, two contact surfaces that form the saddle joint are called saddle-shaped joint surface and M-shaped joint surface respectively, as shown in Figures 2a,b. The third typical surface is the ventral skeletal surface, named as the fan-shaped surface as shown in Figure 2c.

### 2.3 Parameter measurements

**Figure 3:**
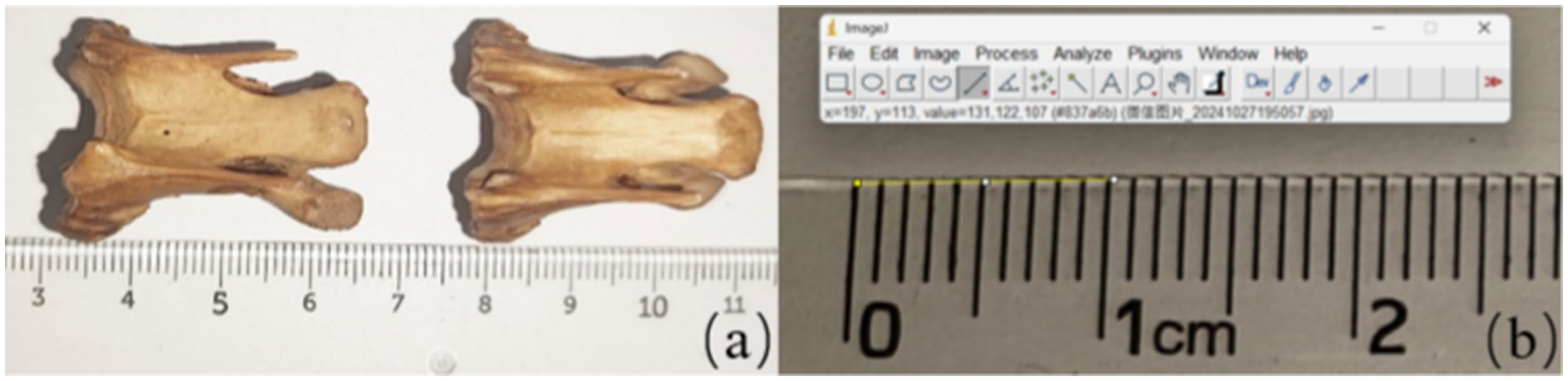
Calibration for measurement method used image J software **(a)** the image taken by camera used to measure **(b)** the unification of measurement tools including ruler and the image J software.

Vernier calipers are accurate and practical tools for size measurement, capable of assessing factors such as length and width. However, due to the complexity of joint construction and mostly irregular features, it is impossible to quantify the specified region parameters using vernier calipers. Therefore, we measured the area parameters of the vertebrae using the Image J software (Version1.8.0, NIH, US). The software is based on a comprehensive array of image processing and analysis tools to support multidimensional data measurement and analysis of digital images. During measurement, calibration of vernier calipers and Image J software are required to ensure the accuracy of both measurement techniques. Calibration of the vernier caliper is done by measuring the standard blocks and comparing the measurements with the known values of the standard quantity block. The calibration process of the Image J software is to place the bone sample and the standard proportion in the same plane and keep the camera height constant. Sample images were taken to ensure that the bone sample and the standard scale appeared in the same photograph. The captured images were imported into the Image J software, using the “Analyze-set scale” tool, calculating the size of each pixel and determining the size of the area to be measured based on the area of per unit pixel during continuous measurement. The calibration is done by comparing the obtained results with the actual values of the sample (see Figure 3).

Measure the VH, ZW, CH, CW, CL of 4 geese C3-C15 and 4 ducks C3-C12 using an electronic vernier caliper (measuring range 0-150 mm). Each parameter was tested three times, and the resulting VH, ZW, CH, CW and CL values were averaged and the standard deviations were calculated to determine the precise structural features of the vertebrae.

**Figure 4:**
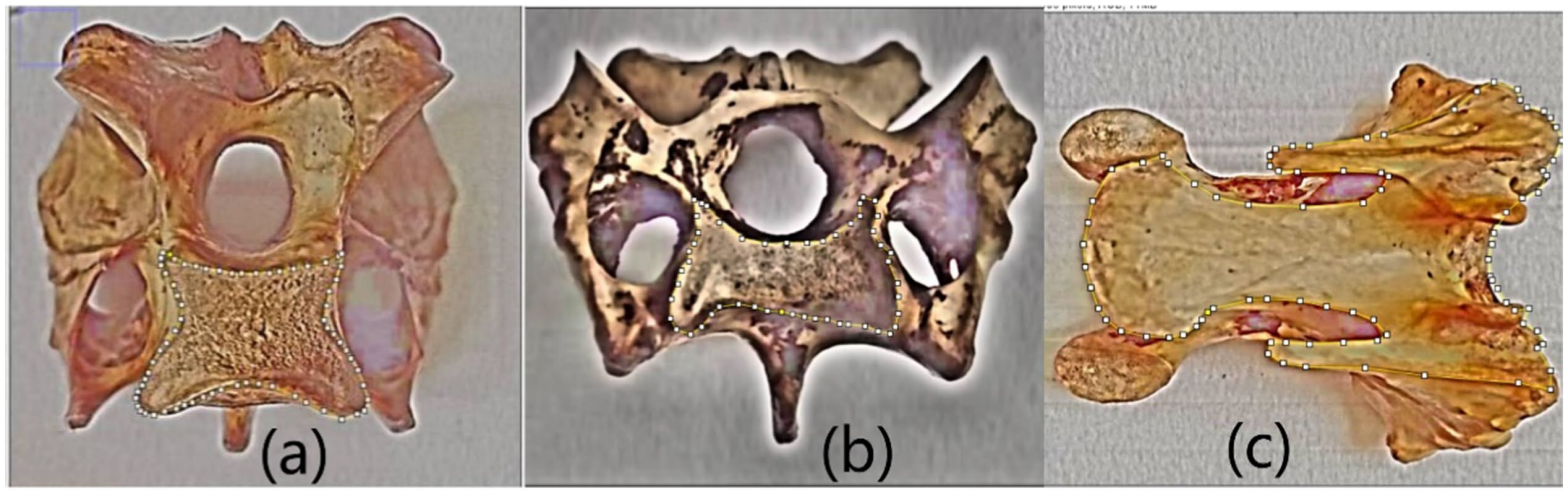
Testing area of three typical vertebral surfaces using ‘Polygon selections’ tool **(a)** Saddle-shaped joint surface **(b)** M-shaped joint surface **(c)** fan-shaped surface.

Moreover, due to the complex shape of bones, their area parameters cannot be accurately measured using vernier calipers, thus using Image J software. The bone specimen was placed under the camera for photography and used to map the area to be measured using Image J software. After drawing, use the “Analyze-set scale” tool to measure the area parameters of the selected area in the image and record the data. As shown in Figure 4.

## 3 Results

**Figure 5:**
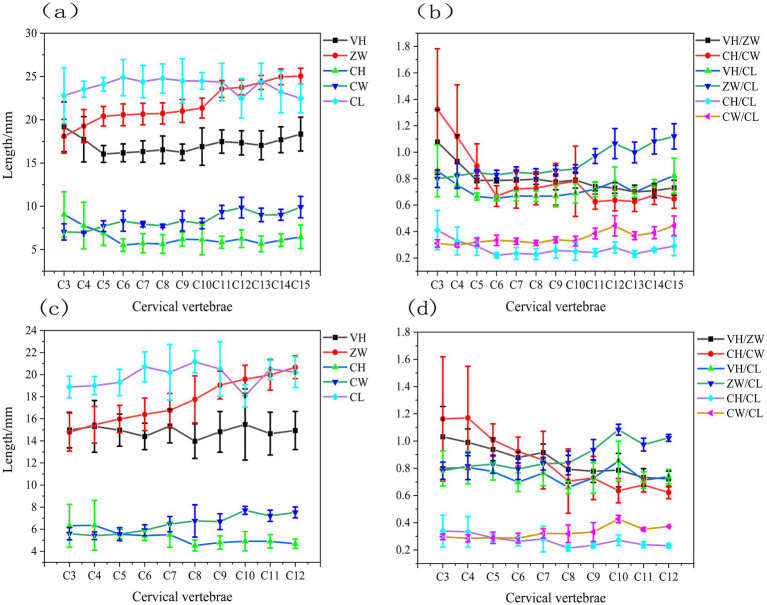
Structural parameters of the gooseneck vertebrae **(a)** the structural parameters including VH, ZW, CH, CW, and CL of geese **(b)** the ratios including VH/ZW, CH/CW, VH/CL, ZW/CL, CH/CL, and CW/CL of geese **(c)** the structural parameters including VH, ZW, CH, CW, and CL of ducks **(d)** the ratios including VH/ZW, CH/CW, VH/CL, ZW/CL, CH/CL, and CW/CL of ducks.

The structural parameters VH, ZW, CH, CW, and CL of the caudal end of the cervical vertebrae were acquired for each vertebra from four geese and four ducks using the aforementioned approach. To further analyze the structural properties of the avian cervical spine, we calculated the ratios of various structural parameters, including VH /ZW, CH / CW, VH / CL, ZW / CL, CH/ CL, and CW/CL. The results obtained are shown in Figure 5.

Figure 5a shows that the VH of geese progressively diminished in the C3-C5 segment, approximated in the C5-C10 section, then rapidly ascended in the C10-C15 segment. The CL progressively grew in the C3-C6 section, remained relatively constant in the C6-C11 segment, then gradually diminished in the C11-C15 segment. The length and height of the geese’s neck vertebrae exhibited inverse patterns; specifically, the height of the vertebrae diminished progressively as the length grew. The value of CH in the geese neck vertebrae progressively diminished from segments C3-C6, was comparable in segment C6-C10, and gradually ascended in segment C10-C15, whereas the value of CW displayed an inverse trend to CH in segment C3-C6 and mirrored the trend of CW in segments C6-C10 and C10-C15. The ZW value of the geese neck vertebrae progressively escalated, with a markedly greater rise observed in the C3-C5 and C10-C15 segments compared to the C5-C10 segments.

Figure 5b shows that the CH/CW values of geese neck vertebrae exhibited a quick decline in the C3-C6 segments, transitioning from CH/CW > 1 to CH/CW < 1, followed by a moderate increase in the C6-C10 segments, and then a reduction in the C10-C15 segments. The VH/ZW values diminished swiftly in the C3-C5 segments, transitioning from VH/ZW > 1 to VH/ZW < 1, were more comparable in the C5-C10 segments, and were markedly greater than those in the C5-C15 portions within the C5-C10 section. The C10 segments were in proximity and progressively diminished in the C10-C15 segments. The VH/CL and CH/CL of the goose neck vertebrae exhibited a similar pattern: a decrease in the C3-C6 segments, convergence in the C6-C10 segments, and an increase in the C10-C15 segments. The geese neck vertebrae ZW/CL and CW/CL exhibited a continuous trend of alteration, namely, a proximity in the C3-C10 segments and a steady increase in the C10-C15 segments.

Figure 5c shows that the VH of duck, exhibited a progressive decline in segments C3-C6, a gradual increase in segments C6-C10, and a subsequent slow decline in segments C10-C12. The CL of duck progressively rose in segments C3-C6, subsequently dropped in segments C6-C10, and then increased again in segments C10-C12. The length and height of duck neck vertebrae exhibited entirely contrasting patterns; specifically, an increase in CL corresponded with a decrease in VH. The value of CH in duck neck vertebrae progressively diminished in the C3-C8 segments and was more comparable in the C8-C12 segments, whereas the value of CW exhibited a tendency to increasing augmentation. The ZW value of duck cervical vertebrae exhibited a progressive and significant rise.

Figure 5c shows that the CH/CW ratios of duck cervical vertebrae diminished swiftly, transitioning from CH/CW > 1 to CH/CW < 1. The VH/ZW values declined sharply, transitioning from VH/ZW > 1 to VH/ZW < 1 in the C3-C6 segments, while decreasing gradually in the C8-C12 portions. The duck cervical vertebrae VH/CL and CH/CL exhibited the same pattern: a progressive decrease in the C3-C6 segments, a gradual increase in the C6-C10 segments, and a subsequent decrease in the C10-C12 segments. However, the amount of change in VH/CL was much larger than that in CH/CL. The duck cervical vertebrae ZW/CL and CW/CL exhibited similar trends, namely in the C3-C16, C6-C10, and C8-C12 segments, which were closer together. The ZW/CL and CW/CL exhibited a consistent trend of variation, specifically converging in the C3-C16 segments, progressively increasing in the C6-C10 segments, and further increasing in the C10-C12 segments. The magnitude of change in ZW/CL was significantly greater than that of CW/CL, with the value of ZW/CL approaching 1 after the C10 segments.

**Figure 6:**
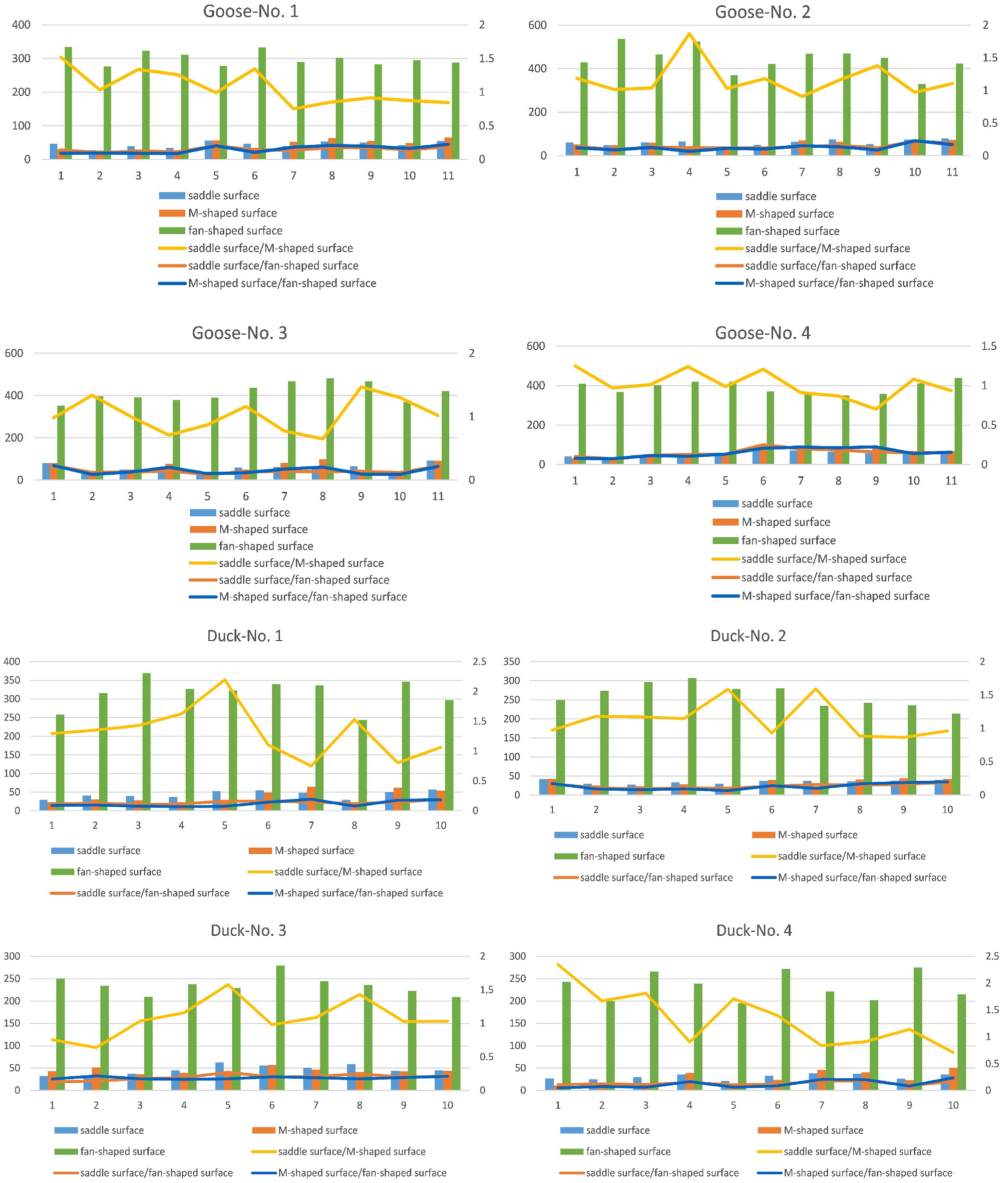
Surface area of the saddle surface (saddle-shaped joint surface), M-surface (M-shaped joint surface), and fan-shaped surface in relation to the ratios of the saddle surface to M-surface and fan-shaped surface to M-surface of four geese (numbered as Googe-No.1, No. 2, No. 3, and No.4) and four ducks (numbered as Duck-No.1, No. 2, No. 3, and No.4).

Structural features of the cervical caudal, saddle-shaped, m-shaped, and fan-shaped articular surfaces of four geese and four ducks are displayed in a uniform histogram. Meanwhile, in order to study the correlation of the motion fit of adjacent joints to the whole surface properties, the ratio of saddle-to m-joints, saddle-and m-joints to sectors is represented by line plot. As shown in Figure 6.

Figure 6 illustrates that among the three typical surfaces, the ventral surface possesses the largest area. This area can be utilized to determine the specific dimensions of a skeleton. The saddle-shaped joint surface and the M-shaped joint surface serve as two connecting surfaces between the two skeletons, exhibiting similar dimensions. The folding diagram demonstrates that, regardless of whether it pertains to geese or ducks, the ratio of the saddle-shaped joint surface to the M-shaped joint surface remains relatively stable, typically approximating 1. The maximal cranial flexion angle between neighboring vertebrae is partly correlated with the ratio of the saddle-shaped joint surface to the M-shaped joint surface. The saddle-shaped surface area for geese and ducks varied from 20 to 40 square millimeters. The ratio of the fan-shaped surface to the saddle-shaped joint surface varied significantly, ranging from 5 to 11. The area of the fan-shaped surface varied from two hundred to five hundred square millimeters, with a significant disparity in the ventral surface across the various skeletons.

The cervical vertebrae of ducks and geese share similar structural features and can be divided into three segments: the cranial segment (C3-C6), middle segment (C6-C10), and caudal segment (C10-C15 in ducks; C10-C12 in geese) (27–29). In the cranial segment, the saddle-shaped joint surface and M-shaped joint surface exhibit nearly identical surface areas, while the smaller fan-shaped surface enhances axial rotation and lateral flexion capabilities. The caudal segment maintains equal surface areas between the saddle-shaped and M-shaped joint surfaces but features an enlarged fan-shaped surface, which reduces axial mobility while improving lateral flexion capacity. In the middle segment, the larger fan-shaped surface and higher ratio of saddle-shaped to M-shaped joint surface areas result in slightly diminished axial and lateral mobility compared to the other segments.

## 4 Discussion

This study employed a measurement method that combines traditional vernier calipers with image processing technology to measure cervical spine specimens from four Chinese domestic geese and four Chinese domestic ducks. It measured cervical morphological parameters (VH, ZW, CH, CW, CL) and the characteristics of saddle-shaped joint surfaces and M-shaped joint surfaces, using the ventral bony surface (fan-shaped surface) as a standardized reference.

For each goose and duck, their cervical skeletal morphology exhibits remarkable similarity in all features except for vertebral count. Given that both domestic geese and ducks are birds adapted to aquatic and terrestrial environments, we hypothesize that this morphological similarity may be related to their dietary and environmental factors. However, Terray et al. (30) demonstrated that dietary and environmental factors can only account for partial shape variations between functional cervical modules. For instance, in this study, the CH parameter occasionally shows an extreme value in domestic geese and ducks, while generally maintaining a relatively narrow fluctuation range. Nevertheless, this cannot fully explain the evolutionary patterns of cervical skeletal morphology. To verify whether dietary and environmental factors are associated with the evolution of cervical skeletal characteristics, it is necessary to incorporate a broader range of avian species into the study for more comprehensive investigation.

Boas (7) divided the avian cervical spine into three main regions based on the degree of dorsal curvature: region 1 where ventral curvature is prevalent, region 2 where dorsal curvature is prevalent, and region 3 where both dorsal and ventral curvature are limited. The boundaries between these areas are given by the transitional vertebrae with a specific morphology.

Based on the dimensional characteristics of the neck bones and the ratio between the saddle, m-shaped and fan surfaces (Figure 5), the cervical spine was divided into three different subdivisions. The results showed that the cranial vertebrae had better axial and lateral flexion capacity of domestic geese and domestic ducks, the caudal vertebrae had less axial mobility but more lateral flexion capacity, and the axial motion capacity and lateral flexion of the middle vertebrae were lower than those of the other two segments. This is consistent with the findings of Kambic et al. (6), Christian and Dzemski (31), Cobley et al. (14), and Virchow (32).

These differences in mobility may be closely related to the life habits of domestic geese and domestic ducks. For example, the high mobility of the cranial vertebrae may facilitate their flexible head rotation while foraging and swimming in the water, and their strong lateral mobility, which may facilitate their balance while walking and turning on land. Moreover, the relatively low degree of activity of the middle vertebrae reflects the stability and supporting role of this region in the overall function of the neck. Further studies could explore the specific associations between neck motion abilities and the behavioral habits of domestic geese and ducks, and the universality and divergence of this structural feature in different birds.

## 5 Conclusion

This study investigated Chinese domestic geese and ducks as research subjects, measuring characteristic parameters of their cervical vertebral morphological structures. Through comparative analysis of vertebral structural features across different cervical regions, we examined the relationship between various structural characteristics and kinematic properties. The results revealed remarkable similarity in vertebral morphology between geese and ducks. Analysis of cervical skeletal parameters demonstrated an inverse trend between cervical vertebral height (CH) and width (CW). The fluctuation range of zygapophyseal width (ZW) increased with vertebral centrum length (CL), with geese and ducks showing essentially consistent ZW fluctuation patterns. Compared to duck cervical vertebrae, goose specimens exhibited smaller overall variations in CL values.

Notably, despite differences in cervical vertebral count between geese and ducks, both species displayed functional regionalization patterns similar to other avian species. The findings indicate that cranial segment vertebrae in domestic geese and ducks possess superior capabilities for both axial rotation and lateral flexion. In contrast, caudal segment vertebrae showed reduced axial mobility but enhanced lateral flexion capacity. The middle segment demonstrated inferior performance in both axial movement and lateral flexion compared to the other two regions.

This study can provide theoretical support for the research of bird cervical bone structure model, and also provide reference for revealing the correlation between bird neck structure characteristics and behavioral habits.

## Data Availability

The original contributions presented in the study are included in the article/supplementary material, further inquiries can be directed to the corresponding authors.
